# Supplementary material for: Deaf children with cochlear implants in Chile: A national analysis of health determinants and outcomes in the Latin American context
Source: PLoS One. 2025 Mar 5;20(3):e0317238. doi: 10.1371/journal.pone.0317238 (PMC11882099; doi:10.1371/journal.pone.0317238)
Supplement: S1 File — (PDF) [file pone.0317238.s001.pdf]

TRASLATION FROM DOCUMENT ETHICS UNIVERSITY OF CHILE

FACULTY OF MEDICINE
UNIVERSITY OF CHILE –
FACULTY OF MEDICINE HUMAN RESEARCH ETHICS COMMITTEE

PROJECT APPROVAL ACT
(Document in version 3.1 corrected 17.11.2020)
On December 1, 2020, the Human Research Ethics Committee of the Faculty of Medicine, University of Chile, composed of the following members:
Dr. Manuel Oyarzun G., Pulmonologist, Associate Professor, President
Dr. Lucia Cifuentes O., Medical Geneticist, Full Professor, Deputy Vice President Ms. Claudia Marshall F., Educator, Community Representative
Dr. Gricel Orellana, Neuropsychiatrist, Associate Professor
Prof. Julieta González B., Cell Biologist, Associate Professor
Dr. María Angela Delucchi Bicocchi, Pediatrician Nephrologist, Associate Professor
Dr. Miguel O'Ryan, Infectious Disease Physician, Associate Professor
Dr. María Luz Bascuñán Psychologist PhD, Associate Professor
Ms. Karima Yarmuch G., Lawyer, Legal Directorate, Faculty of Medicine
Ms. Javiera Cobo R., Nutritionist, Executive Secretary
Prof. Verónica Aliaga C., Kinesiologist, Master in Bioethics, Associate Professor
Dr. Dante Cáceres L., Veterinarian, Doctor of Public Health, Associate Professor

He has reviewed the Research Project entitled: "CHARACTERIZATION OF COCHLEAR IMPLANT USERS BENEFICIARIES OF THE GES, RICARTE SOTO LAW AND COCHLEAR IMPLANT PROGRAM OF THE MINISTRY OF HEALTH". Its principal investigator is Prof. Mario Bustos, who works in the Department of Speech and Language Therapy, Faculty of Medicine, University of Chile.

The Committee reviewed the following study documents:
Research Project
1. Researcher's CV
    1. Informed Consent
Letter of commitment from the researcher to communicate the results of the study once the study is completed
The project and the documents mentioned in the previous paragraph have been analysed in the light of the postulates of the Declaration of Helsinki, the International Ethical Guidelines for Biomedical Research on Human Beings CIOMS 2016, and the ICH 1996 Guidelines for Good Clinical Practice.
Phone: 29789536
-

Based on this information, the Human Research Ethics Committee of the Faculty of Medicine of the University of Chile has pronounced itself as follows on the aspects of the project that are indicated below:
a) Nature of the population to be studied (captive/non-captive; therapeutic/non-therapeutic research): Pediatric and adult beneficiaries of the Ricarte Soto Law and GES, and of the MINSAL program for the implementation of cochlear implants in patients with severe/profound hearing loss. It is retrospective, captive and non-therapeutic.
b) Usefulness of the project: Very relevant study, requested by MINSAL.
c) Risks and benefits: No risks greater than minimal are visualized.
d) Protection of participants (ensured by Informed Consent): Yes.
e) Timely notification of adverse reactions: Not applicable.
f) Commitment of the responsible researcher to notify the results of the study at the end of the project: Yes.
g) Requires follow-up Field visit: If No_ X
No. of views:

Therefore, the committee considers that the proposed study is well justified and that it does not pose any physical, psychological or social risks greater than minimal for the subjects involved.

This committee also analyzed and approved the corresponding Informed Consent documents in their modified version received on November 26, 2020, which is attached signed, dated and stamped by this CEISH.
Notwithstanding the foregoing, in accordance with the provisions of Article 10 bis of Supreme Decree No. 114 of 2011, of the Ministry of Health, which approves the regulations of Law No. 20,120; it should be remembered that all scientific research on human beings must have the express authorization of the director or directors of the establishments within which it is carried out, which must be carried out within 20 working days from the conformity evaluation of the CEISH, being the responsibility of the researcher to send this Committee a copy of it within the indicated period.
Teléfono: 29789536 - Email: comiteceish@med.uchile.cl

UNIVERSITY OF CHILE
ETHICS COMMITTEE FOR RESEARCH ON HUMAN BEINGS
FACULTY OF MEDICINE
01 0102120
