# Supplementary material for: Deaf children with cochlear implants in Chile: A national analysis of health determinants and outcomes in the Latin American context
Source: PLoS One. 2025 Mar 5;20(3):e0317238. doi: 10.1371/journal.pone.0317238 (PMC11882099; doi:10.1371/journal.pone.0317238)
Supplement: S2 File — (DOCX) [file pone.0317238.s002.docx]

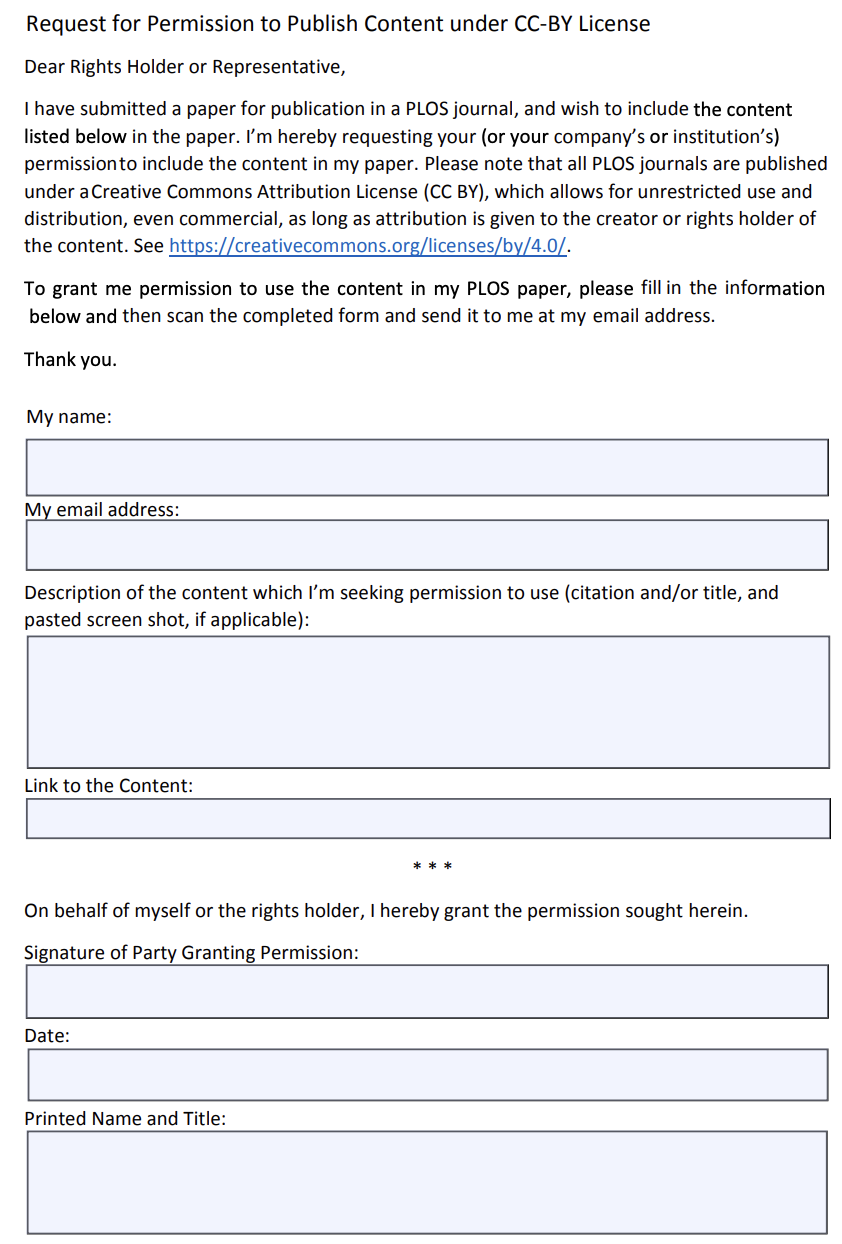


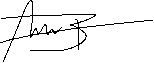


Mr. Mario Bustos-Rubilar

PhD Psychology and Language Sciences

Publication: Deaf children with cochlear implants in Chile: A national analysis of health determinants and outcomes in the Latin American context

15 November 2024

MARIO BUSTOS RUBILAR

I request permission for the open-access journal PLOS ONE to publish “Figure 1. Geographical distribution of deaf children with CI (N=107) by BDI in Chile” in the publication “Deaf children with cochlear implants in Chile: A national analysis of health determinants and outcomes in the Latin American context.” under the Creative Commons Attribution License (CCAL) CC BY 4.0 (http://creativecommons.org/licenses/by/4.0/). Please be aware that this license allows unrestricted use and distribution, even commercially, by third parties. Please reply and provide explicit written permission to publish “Figure 1. Geographical distribution of deaf children with CI (N=107) by BDI in Chile” in the publication “Deaf children with cochlear implants in Chile: A national analysis of health determinants and outcomes in the Latin American context” under a CC BY license and complete the attached form

Zczl085@ucl.ac.uk

Mario Bustos-Rubilar
