# Supplementary material for: Deaf children with cochlear implants in Chile: A national analysis of health determinants and outcomes in the Latin American context
Source: PLoS One. 2025 Mar 5;20(3):e0317238. doi: 10.1371/journal.pone.0317238 (PMC11882099; doi:10.1371/journal.pone.0317238)
Supplement: S4 File — Appendix 2. Informed consent. Appendix 3. Protocols. Appendix 4. Analysis of total cost of CI in Chile. (DOCX) [file pone.0317238.s004.docx]

**Deaf children with cochlear implants in Chile: A national analysis of health determinants and outcomes in the Latin American context.**

Mario Bustos-Rubilar^1,2,*^, Fiona Kyle^1^, Merle Mahon^1^

*^1^ Division of Psychology and Language Science, University College London, London, UK*

*^2^ Departamento de Fonoaudiología, Facultad de Medicina, Universidad de Chile, Santiago, Chile.*

^*^ **Corresponding author**. Division of Psychology and Language Sciences, Brain Faculty, University College London, 2 Wakefield St, London WC1N 1PJ, email: [mario.rubilar.18@ucl.ac.uk](mailto:mario.rubilar.18@ucl.ac.uk)

​​**Table of Contents**

​Appendix 1 – Consort Diagram for participant selection

Appendix 2- Informed Consent

Appendix 3 – Protocols

Appendix 4 – Analysis of total cost of CI in Chile

**Appendix 1 – Consort Diagram for participant selection**


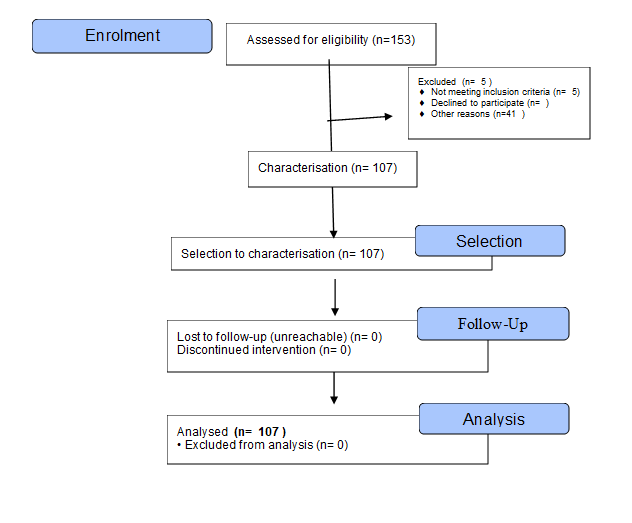


+

**Appendix 2- Protocols.**

1. Informed Consent for parents/caregivers of deaf children with CI

**Factors affecting outcomes in Deaf or Hard Hearing Children with Cochlear Implant in Chile considering the Latin-American context.**

(This study has been approved by the UCL Language and Cognition Department Ethics Chairs, project number LCD-2020-13 and The Faculty of Medicine, University of Chile project number 167-2020)

Thank you for agreeing to continue to take part in this study about factors affecting spoken language development in children with CI in Chile. We appreciate your participation again in this survey after one year.  We have shortened the survey we used last time. You should need now around 20 minutes for completing it.  Please be sure that the person who responded last time also fills in the survey this time. You need to complete again each question about you and/or your child. It is strongly recommended that you complete the survey at one time. Some problems or loss of information could occur if you leave the survey uncompleted.

We need to get your informed consent again, giving specific details about your participation this second time. Please complete the information about your bank account at the end of the survey so that we can compensate you for taking part. Please read the following information and, if you agreed, completed the consent again at the end of this text. Thank you again for your participation!

**Informed Consent:**

*You are reading the information sheet for collecting data through this survey. The purpose of this information is to help you decide for you and your child to continue to participate in the research. "*

***The research objective*** *is to characterise the Chilean cochlear implant users from Public System Health implanted from 2018 and to investigate factors that affect children’s language development.*

*If you agree to participate, we will invite you to take part in a survey to ask you about the characteristics of your family and your child. The survey will once more be through OPINIO online platform, or if you need it, through telephone assistant.  This will take about 20 minutes. We will collect information about your family, your child, their cochlear implant, their language used, their treatment and the education they received.*

*The information collected will be confidential and pseudonymised, according to Chilean and British law (Data Protection Act 2018) about protecting user data. It means that real names will not be used, and your child and this interview will have an ID number. Nobody in your Hospital will know any answer you give today. Only the research team will have access to the data.*

*The data will be analysed at University College London in the UK. All data transferred to the UK will be fully anonymised, and no personally identifiable data will be transferred to the UK.*

***About benefits*** *– The results obtained will be useful to our understanding of the characteristics of cochlear implant users in the Chilean public system. Additionally, the information will contribute to improvement to the current cochlear implant programs in Chile.  If required, at the end of the study, we can send you a summary report of the study results.*

***About risks*** *- This research has no harmful effects.*

***About compensations*** *– There will be 6200 Chilean pesos of compensation for your invested time in the survey filling. At the end of the survey, we will ask you for your bank account details. The deposit will be paid two weeks after you have completed the survey.*

***This will be voluntary participation*** *- Your participation in this research is entirely voluntary. It is up to you to decide whether or not to take part. If you choose not to participate, you won't incur any penalty or loss of benefits to which you are otherwise entitled as a patient at the Hospital. Refusing will not affect the quality of care you or your child deserves.*

*You need to add in the* ***Data Protection Privacy Notice****asset out in the information sheets in your original ethics application.*

***Questions*** *- If you have questions about your rights as a participant in medical research, you can contact with the principal research of this study.*

***Name and Contact Details of the Researcher(s):***

*The principal research is Mario Bustos Rubilar, his mail* [*mario.rubilar.18@ucl.ac.uk*](mailto:mario.rubilar.18@ucl.ac.uk) *telephone 022-9786606.*

***Please fill in this informed consent form if you agree to participate:***

| YES / NO | *I confirm that I have read and understood the Information Sheet for the above study. |
| --- | --- |
| YES / NO | *I understand that I will be able to withdraw my data |
| YES / NO | *I consent to participate in the study. I understand that my personal information will be anonymised and used according to the aforementioned policies. |
| YES / NO | *I understand that my participation is voluntary and that I am free to withdraw at any time without giving a reason. |
| YES / NO | I understand the direct/indirect benefits of participating. |
| YES / NO | I understand that I will be compensated for the time spent in the study |
| YES / NO | I agree that research data may be used by others for future research. |
| YES / NO | I am aware of who I should contact if I wish to lodge a complaint. |
| YES / NO | I voluntarily agree to take part in this study. |
| YES / NO | I confirm I could be contacted at the future for a further research. |

**Appendix 3  - Protocols**

This Interview Protocol Child with CI Survey 1 (Survey C1) was completed previous the translation and adaptation to Chilean Spanish. Some questions and options were slightly changed.

**PART 1** - INSTRUCTIONS AND SUMMARY SHEET

TELEPHONE INTERVIEW USING OPINIO PLATFORM

INSTRUCTIONS

This document must be used only by trained interviewers. This protocol has five sections:

- **Part 1** - Instructions and Summary Sheet
- **Part 2** - Informed Consent
- **Part 3** - Missing Data from Clinical Record
- **Part 4** - Data About Auditory and Language Abilities
- **Part 5** - Factors Data

**The** interview must follow the following steps as per the training *(For any question, you can check the interview instructions given in the training):*

1. Interviewer presentation and credentials.
2. Establish rapport with parents/caregivers giving details about CI user (Information about cochlear implant and Hospital).
3. Project presentation and digital link for additional information (Trust about the study).
4. Ask for the parent/caregiver closest to the CI user and request her/his participation.
5. Explain the interview, giving aim, duration of the interview and need for informed consent.
6. Read the information sheet and consent form and request his/her positive answer saying "Yes" and their complete name.
7. To deliver the protocol, i.e. ask the questions
8. Record the interview.
9. We are considering extra time for open-ended questions (Qualitative Data) in *Italic letters.*
10. Remember, do not read the titles of any section.
11. Remember, do not pre-empt an answer (Interview instructions).

PARENTS/CAREGIVER INFORMATION:

*(Information need to be completed by the interviewer for cross-check information)*

Name: _____________________________________________________________________.

Hospital: ___________________________________________________________________.

Phone/Mail: ________________________________________________________________.

ÿ Informed Consent given.

ÿ Interview Protocol completed.

CI USER IDENTIFICATION

**Personal Information about the CIu** *(TCMH will provide this information)*

Anonymized ID:  ________________.         Date of Birth: ___________________________.

Hospital: ______________________.       Date of Switch on: ________________________.

**Missing crucial data from the clinical record, which need to be collected (PART 3):**

*(The researchers will previously mark this information)*

ÿ Personal Information _______________________________________________________.

ÿ Family Information _________________________________________________________.

ÿ Audiological Information ____________________________________________________.

ÿ Medical History ____________________________________________________________.

ÿTreatment Information _______________________________________________________.

OBSERVATIONS (Issues, pending action, missing data, etc.).

___________________________________________________________________________

___________________________________________________________________________

___________________________________________________________________________

**PART 3** - MISSING DATA FROM CLINICAL RECORD

**1. TELEPHONE INTERVIEW QUESTIONS FOR PARENTS/CAREGIVERS OF CI USERS.**

**1.1 Personal Information:**

1. Child's age: years______ months _____
2. SES / FONASA: A___. B ____. C____. D____.
3. Address: __________________________________________________________________
4. Household composition: (* e.g. Nuclear family, only one parent, other, etc.)
5. _____________________________________________________________________________________________________
6. Mother/Caregiver Education Level ( *e.g. Unknown, primary school, high school, etc.)

__________________________________________________________________________

**1.2 Medical and Audiological Information:**

1. Additional Difficulties (*e.g. No known, Cerebral palsy, Learning difficulties, Psychomotor delay, etc.)

_____________________________________________________________________________

1. Medical History (*e.g. None, Grommets, Jaundice, Epilepsy, etc.)

_____________________________________________________________________________

1. Family History (*e.g. None, Hearing impairment, cognitive condition, etc.)

_____________________________________________________________________________

1. Date of birth _____________________                                (Age of Diagnosis: _____ Months).
2. Age at first hearing aid issue: _____ Months
3. Age at CI implantation _____ Months.
4. Age of Switch on (CI)    _____ Months.
5. Implant and hearing aid info (*e.g. Unilateral with or without Hearing aid, sequential implant, etc.)
6. Before of the CI, Did He/she use the hearing aid: freqtly____ s/times _____ rarely ____
7. CI condition now (*e.g. No problems, CI without use, technical issues, etc.)

1. **Additional Information about treatment and language used** (Mandatory field)
2. Is your child attending auditory or language therapy?                                        Y____ N____
3. Where (Therapy): ___________________________________________________________
4. Is the therapy Weekly, Monthly? _______________________________________________
5. How much time do you spend in each session?      30 min____ 40min____ 60min or +____
6. Is it easy for you to get to the sessions? Yes___ No___ Why? (*) ______________________
7. Is it easy for you follow the guides given by the CI Staff? Y____ N____ Why? (*) __________________________________________________________________________
8. What communication mode is used in therapy:

 sign lang ___, spoken lang ___, both mixed___, both separate ___.

1. Does your child attend nursery or school?  Y____ N____. Where? ____________________.
2. How frequently does he/she attend the school/nursery?

How many days per week? _______.  How many hours per day? 2h___ 4h____6___8or+___.

1. What type of school/nursery is it special education/ mainstream with PIE / mainstream with no special need support? (e.g. examples if they require them) _____________________________.
2. If he/she receives support for special need, who delivers this support? (e.g. therapist, interpreter, special education)

      ÿ Interpreter  ÿSLT  ÿ Audiologist  ÿ S. needs Teacher ÿ AVT Therapist ÿ Psychologist

1. Did your child attend to auditory or language therapy before the current treatment? Y_ N_

**PART 4** - DATA ABOUT AUDITORY AND LANGUAGE ABILITIES

**2. AUDITORY AND LANGUAGE ABILITIES IN THE CHILD**

2.1 COMMUNICATION

1. How do you communicate with your child?

ÿ Sign L  ÿSpoken L ÿ Mixed L  ÿ Pre-Formal Com (e.g. as baby)  ÿ Other ___________________

2. Can s/he communicate with unfamiliar people?                                                                 Y____ N____

How (*) __________________________________________________________________________

3. Do your child use sign language at home/in the school/ or with friends?                      Y____ N____

4.  CAP AND SIR

 Please tell me if your child can do the following actions at home with the CI:

**CAP**

| **YES /NO** | **Action (Possible questions for each scale step)** |
| --- | --- |
|  | **Does** *your child react from the sound in the house or outside? For example, the doorbell, the dump truck, the sound from the TV?)* |
|  | Do your child show a response to speech sounds such as /a/, /m/, /s/, /sh/ i/e/ /o/n the LST session or with you at home? |
|  | Can your child identify some sounds from the house, pointing for example; the Doorbell, the door closing, the telephone ringing, animals (Such as a dog or cat)? |
|  | Can your child discriminate between two sounds (such as hearing the sound ‘woof woof’ and pointing to/looking at the dog vs hearing the sound ‘quack quack’ and pointing to the duck, or his/her name vs some sound. |
|  | Can your child follow a short instruction only by using the hearing? (For example, sit here, where is papa? collect your toys). |
|  | Can your child maintain a conversation with some people familiar without the use of lip reading? (For example, with some uncle or aunt, or his/her teacher?). |
|  | Can use the telephone with a familiar talker? |
|  | Can understand/follow group conversations? For example, in school? Or family meetings? |
|  | Can use the telephone with an unfamiliar person or unfamiliar topic/context? |

5.   **SIR:** Now, please tell me **how does your child communicate?**

| **YES /NO** | **Action (Possible prompt questions for each scale step)** |
| --- | --- |
|  | 1. Is your child communicating using gesture/pointing and some vocalisations?   (Pre-recognisable words in spoken language (the child's primary mode of everyday communication may be manual) |
|  | 1. Is your child using any words that you recognise?   (Connected speech is unintelligible; intelligible speech is developing in single words when context and lip-reading cues are available) |
|  | 1. Can you understand your child’s spoken Phrases/sentences when you are focused, you see the background, and you can lip-read?   (Connected speech is intelligible to a listener who concentrates and lip-reads within a known context). |
|  | 1. Can you and others who do not know your child understand what s/he says?   (Connected speech is intelligible to a listener who has little experience of a deaf person's speech; the listener does not need to concentrate unduly). |
|  | 1. Do your child is understood easily in everyday contexts?   (Connected speech is intelligible to all listeners) |

**PART 5** – FACTORS DATA

**3. FACTORS AFECTING THE SPOKEN LANGUAGE IN CI USERS.**

3.1 CHILD’S COMPLIANCE WITH THE CI

1.  Does your child use the Cochlear Implant?                                                                     Y____ N____.

2.  If the answer is no, Why? __________________________________________________________.

     If the answer is Yes:

1. How many days per week, your child uses the CI?

     2 or less days_____   4 or less days _____ 6 or less days____ All Days______

1. In the current time, how many hours a day does your child currently wear the aid(s)?

     Monday-Friday from_____ to_____ hrs   Saturdays/Sundays from ______ to ______ hrs.

1. When your child wakes from sleep (morning or nap), how much *effort do you* exert to help him/her with the switch on of the device?

Excessive effort___ Some Efforts___ Normal___ No effort ___.

1. Do you agree about this statement; "My child decides if she/he uses or does not use the device during the day". Y__ N__ Why? (*)___________________________________________________.

7. Are there any particularly challenging times or situations for you or the child for keeping the CI? (*)

8. If s/he does not use the device, what do you think is missing and how could this be improved? (*)

__________________________________________________________________________________

3.2. BEHAVIOUR ABILITIES IN CIU

1. Has your child been diagnosed with any behaviour issues?                                          Y____ N ____

(*e.g. behavioural problems, ADHD (Attention Deficit and Hyperactivity Disorder), Oppositional defiant disorder, etc).

_______________________________________________________________________________

1. Do you believe she/he currently has some behavioural problem, which need to be diagnosed?

  Y____ N____

3.    In the case of “Yes”. Are these conditions affecting the use of the CI in your child?    Y____N____

Why? (*) _______________________________________________________________________

3.3 SELF-ESTEEM AND WELLBEING

1. Is your child happy in the school/Nursery?                                     Likert (Unhappy 0 to very happy 5 )

1. Do you feel that your child is included in school and social life?                                         Y____ N____

3. How many good friends has your child got?                                               None__ 1 or 2___ 3 or +____

4. Some of them are deaf?                                                                                                             Y____ N____

5 Does S/He have contact with some deaf activity or community?                                        Y____ N____

*If the answer is not, Why?* ____________________________________________________________

3.4. PARENTS/CAREGIVERS ENGAGE/KNOWLEDGE

1. Did you receive any education or training about hearing loss, devices or language development after the diagnosis of your child? Y____ N____.

 What topic was considered? (*)_______________________________________________________.

1. Who gave you that training?

Doctor___ Audiologist___ SLT___ SEN___ Other: ____  Where?______.

1. Did you receive specific training about CI (e.g. Device use, care of the device, etc)  Y____N____
2. That training was:

During the child’s session____  session only for this_____ written documents/instruction_____

6. How many sessions did you receive this training:        1 or 2_____ 2 to 4_____ more than 4 ______

7. Who have you guided you with instructions about the CI in your child? _______________________

8. Do your feel confident with the knowledge that you have about the CI?

Likert (Poor confident 0 to very confident 5 )

9. Do you follow instructions given by professionals?     All of them____ some of them____ No _____

10. Why? __________________________________________________________________________

11. Do you think that you have enough time for working in the rehabilitation process of your child?

Y___ N___

 If it is not, why? ____________________________________________________________

12. If you receive some homework for your child with the CI. How many hours do you work on it?

No work in home ____ I do not set aside specific time for that _____     1____ 2____ 3_____ 4 or more ______

13. How do you check if the Cochlear Implant is working? ___________________________________

14. Has the use of the device satisfied your expectations as a parent/caregiver?

High satisfied____ Satisfied ____ No Satisfied ____ unsatisfied____.

If the answer is not, why? (*)__________________________________________________________

16. What recommendation would you give to:

 the CI Staff?(*): ____________________________________________________________________

 the nursery/School?(*): _____________________________________________________________

**This is the end of this interview. Thank you very much.**

**S4 Appendix – Analysis of total cost of CI in Chile**

As an exploratory analysis, we collected data on the devices, treatment and rehabilitation costs of the CI intervention of deaf children based on expert knowledge and the Chilean public national central medical store (www.cenabast.cl) and data from the Technical Regulation for CI provision in children GES 77 in the following table:

Table S4. Table of costs for cochlear implant treatments among adult patients in Chile

| **Item** | **1^st^ year** | **3^rd^ and 4^th^ year** | **4^th^ and 5th year** |
| --- | --- | --- | --- |
| Cochlear Implant Standard Kit | USD$21100 (CLP$ 18990000) | NC | NC |
| Surgery | USD$3500 (CLP$ 3150000) | NC | NC |
| Hospital additional costs | USD$3200 (CLP$ 2880000) | NC | $USD 1000 (CLP$ 900000) |
| Medical exams | USD$1270 (CLP$ 1143000) | $USD220 (CLP$ 198000) | $USD220 (CLP$ 198000) |
| Medical appointments after surgery | USD$300 (CLP$ 270000) | $USD300 (CLP$ 270000) | $USD300 (CLP$ 270000) |
| Audiological appointments | USD$160 (CLP$ 144000) | $USD220 (CLP$ 198000) | $USD220 (CLP$ 198000) |
| Calibration and training sessions | USD$1500 (CLP$ 1350000) | USD$2800 (CL$ 2520000) | USD$2800 (CL$ 2520000) |
| Accessories replacement (Processor after five years) | NC | NC | USD$12000 (CLP$ 10800000) |
| Transport to the health centre | USD$200 (CLP$ 180000) | USD$400 (CLP$360000) | USD$400 (CLP$360000) |
| Other costs (Legal documents, personal care supplies, etc.) | USD$300 (CLP$270000) | USD$40 (CL$36000) | USD$40 (CL$36000) |
| **Total, by year(s)** | USD$31530 (CLP$16730000) | USD$3980 (CLP$ 3582000) | 17040 |
| **Total** | USD$ 52550 (CLP$23010000) | |  |

Notes: The costs are presented in approximated USD$ and (CLP$), rate exchange USD$1=CLP$900 (July 2024). Abb: NC= Not covered. The prices are based the public sector with interventions up to 5 years after the implantation. Source: Our research in hospitals from the Metropolitan Region of Chile, Bio Bio Region and Public National Medical Store CENABAST [www.cenabast.cl](http://www.cenabast.cl) .
