# Supplementary material for: Deaf children with cochlear implants in Chile: A national analysis of health determinants and outcomes in the Latin American context
Source: PLoS One. 2025 Mar 5;20(3):e0317238. doi: 10.1371/journal.pone.0317238 (PMC11882099; doi:10.1371/journal.pone.0317238)
Supplement: S5 File — (DOCX) [file pone.0317238.s005.docx]

**Figure 1.** Geographical distribution of deaf children with CI (N=107) by BDI in Chile


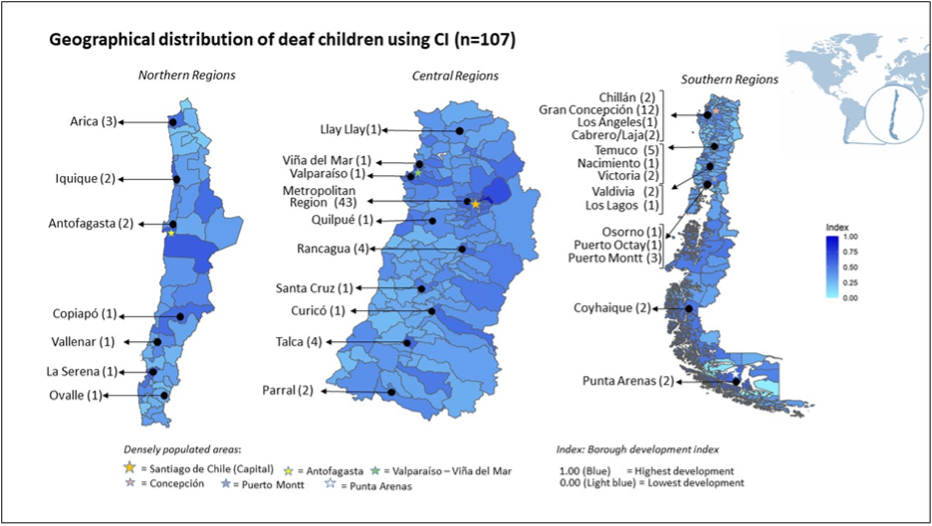


Notes: The colour scale show the Borough Develop Index (25) which evaluates the living environmental deprivation areas within the country. It merges thirteen health, social well-being, economy, and education variables in indexes from 0 to 1. Less developed boroughs are coloured in light blue, while more developed boroughs are in dark blue. The map represents the total of 346 boroughs in the country.
