# Supplementary material for: Deaf children with cochlear implants in Chile: A national analysis of health determinants and outcomes in the Latin American context
Source: PLoS One. 2025 Mar 5;20(3):e0317238. doi: 10.1371/journal.pone.0317238 (PMC11882099; doi:10.1371/journal.pone.0317238)
Supplement: Attachment — Submitted filename: Rebuttal letter Manuscript BustosM et al 2024.docx [file pone.0317238.s006.docx]

**Dear Editor,**

Thank you very much for the opportunity to publish in your journal. We also appreciate the thorough revisions provided by our colleagues and editors members. We have addressed each of the points you and the reviewers raised in accordance with PLOS ONE's requirements for our manuscript titled *"Deaf Children with Cochlear Implants in Chile: A National Analysis of Health Determinants and Outcomes in the Latin American Context."*

Please find our responses below:

**1. Manuscript Style Requirements**
We have reviewed the requirements and edited the style according to the guidelines, as shown in the tracked changes document.

**2. Inclusivity Questionnaire**
The inclusivity questionnaire is attached to the revised manuscript.

**3. Funder’s Role**
We have added the required statement on page 22:
*"The funders had no role in study design, data collection and analysis, decision to publish, or preparation of the manuscript."*
Funding: Data collection was partially supported by a grant from the Chilean Ministry of Health through the National Public Tender 757-89-l120. This article was also supported by a scholarship from the Chilean Government, "Beca de Doctorado en el Extranjero Becas Chile, Convocatoria 2018, Ley N°21.053, Asociación Nacional de Investigación y Desarrollo (ANID)," and the Chilean Ministry of Health through National Public Tender 757-89-l120.

**4. Funding-Related Text**
We have removed any funding-related text from other sections of the manuscript.

**5. Data Availability**
Data is now available in our original project registration form. The following information has been added on page 22:
*Data availability: Data is available to other researchers on The Center for Open Science (COS) at the following link:* [*https://osf.io/4hfd9/*](https://osf.io/4hfd9/)

**6. Ethics Statement**
We deleted the additional ethics statement from page 22, leaving it only in the Methods section.

**7. Copyrighted Figures**
We have attached a document with the required permission for copyrighted images as part of the new submission.

**8. Supporting Information Captions**
We reviewed and added captions for the supplemental materials on page 22.

**9. Reference List**
We reviewed the reference list and confirmed that no cited papers have been retracted.

**Reviewer #1 Comments:**

**10. General Feedback**
Thank you very much for your encouraging words.

**11. Discussion on Cost Analysis**
We agree that the cost analysis section was excessive. We have moved this discussion to the supplemental materials (Appendix 4), mentioning it only briefly as a reference in the main text.

**12. Sample Size Discussion**
We have included a comment on sample size limitations in the last paragraph of the discussion (lines 454-457).

**13. Conclusion Emphasis**
We have revised the conclusion to emphasize the main take-home message (lines 474-481).

**14. Use of ‘Aetiology’**
We have changed "aetiology" to "HL Presentation" in Tables 2 and 3, as suggested.

**Reviewer #2 Comments:**

**15. General Feedback**
Thank you very much for your consideration.

**16. Cochlear Implant Program Description**
We have added a description of the cochlear implant program and selection criteria in paragraph 5 of the introduction (lines 51-54).

**17. Geographic Distribution of Patients**
We have clarified the information regarding the locations of public hospitals authorized for cochlear implant surgery in paragraph 6 (lines 56-59).

**18. Newborn Hearing Screening**
We added this information to the introduction (line 59) and the Methods section, under Population (line 108).

**19. Exclusion of Private Institution Cases**
We clarified the exclusion criteria in the Methods section, under Population (lines 111-114).

**20. Socioeconomic Health Insurance**
We added a description of socioeconomic health insurance classification in Table 1.

**21. Discrepancies in Speech Perception Ratings**
We have discussed the discrepancy in parental vs. clinical ratings in the discussion section, paragraph 7 (lines 397-405).

**22. Confidence Intervals**
We have included confidence intervals in Table 4.

**23. Out-of-Place Table in Discussion**
We have addressed this in our response to point 11 above.

**24. Grammar Revisions**
We have addressed the suggested grammar improvements on pages 10, 11, and 14.

Finally, as a grammatical improvement, we have changed the term hearing loss to deafness to make it simpler and more understandable for any reader, while supporting the reduction of potential ableist terminology when referring to people with disabilities.

Once again, we greatly value all your suggestions, which have helped us improve our manuscript.

Thank you very much, and best wishes,

Mario Bustos-Rubilar et al.
